# Is there a Common Genetic Basis for Autoimmune Diseases?

**DOI:** 10.1080/17402520600876762

**Published:** 2006

**Authors:** Juan-Manuel Anaya, LuisMiguel Gómez, John Castiblanco

**Affiliations:** 1 Cellular Biology and Immunogenetics Unit Corporación para Investigaciones Biológicas Medellín Colombia; 2 Universidad del Rosario Medellín Colombia

## Abstract

Autoimmune diseases (ADs) represent a diverse collection of diseases in terms of their demographic profile and primary clinical manifestations. The commonality between them however, is the damage to tissues and organs that arises from the response to self-antigens. The presence of shared pathophysiological mechanisms within ADs has stimulated searches for common genetic roots to these diseases. Two approaches have been undertaken to sustain the “common genetic origin” theory of ADs. Firstly, a clinical genetic analysis showed that autoimmunity aggregates within families of probands diagnosed with primary Sjögren's (pSS) syndrome or type 1 diabetes mellitus (T1D). A literature review supported the establishment of a familiar cluster of ADs depending upon the proband's disease phenotype. Secondly, in a same and well-defined population, a large genetic association study indicated that a number of polymorphic genes (i.e. *HLA-DRB1*, *TNF* and *PTPN22*) influence the susceptibility for acquiring different ADs. Likewise, association and linkage studies in different populations have revealed that several susceptibility loci overlap in ADs, and clinical studies have shown that frequent clustering of several ADs occurs. Thus, the genetic factors for ADs consist of two types: those which are common to many ADs (acting in epistatic pleitropy) and those that are specific to a given disorder. Their identification and functional characterization will allow us to predict their effect as well as to indicate potential new therapeutic interventions. Both autoimmunity family history and the co-occurrence of ADs in affected probands should be considered when performing genetic association and linkage studies.


*Clinical & Developmental Immunology*, June–December 2006; 13(2–4): 185–195

### Is there a common genetic basis for autoimmune diseases?

JUAN-MANUEL ANAYA[1,2], LUISMIGUEL GÓMEZ[1], & JOHN CASTIBLANCO[1]

[1]*Cellular Biology and Immunogenetics Unit, Corporación para Investigaciones Biológicas, Medellín, Colombia, and*
[2]*Universidad del Rosario, Medellín, Colombia*

**Abstract**

Autoimmune diseases (ADs) represent a diverse collection of diseases in terms of their demographic profile and primary clinical manifestations. The commonality between them however, is the damage to tissues and organs that arises from the response to self-antigens. The presence of shared pathophysiological mechanisms within ADs has stimulated searches for common genetic roots to these diseases. Two approaches have been undertaken to sustain the "common genetic origin" theory of ADs. Firstly, a clinical genetic analysis showed that autoimmunity aggregates within families of probands diagnosed with primary Sjögren's (pSS) syndrome or type 1 diabetes mellitus (T1D). A literature review supported the establishment of a familiar cluster of ADs depending upon the proband's disease phenotype. Secondly, in a same and well-defined population, a large genetic association study indicated that a number of polymorphic genes (i.e. *HLA-DRB1*, *TNF* and *PTPN22*) influence the susceptibility for acquiring different ADs. Likewise, association and linkage studies in different populations have revealed that several susceptibility loci overlap in ADs, and clinical studies have shown that frequent clustering of several ADs occurs. Thus, the genetic factors for ADs consist of two types: those which are common to many ADs (acting in epistatic pleitropy) and those that are specific to a given disorder. Their identification and functional characterization will allow us to predict their effect as well as to indicate potential new therapeutic interventions. Both autoimmunity family history and the co-occurrence of ADs in affected probands should be considered when performing genetic association and linkage studies.

**Keywords:** *Sjögren's syndrome, Type 1 diabetes mellitus, rheumatoid arthritis, systemic lupus erythematosus, genetics, inheritance patterns*

### Introduction

Autoimmune diseases (ADs) are chronic conditions initiated by a loss of immunological tolerance to self-antigens. The chronic nature of such diseases has a significant impact in terms of the utilization of medical care, direct and indirect economic costs and quality of life. The estimated incidence of ADs is about 90 cases per 100,000 person-year and their prevalence is about 3% of the population (Cooper and Stroehla 2003). Almost all ADs disproportionately affect middle age-women and are among the leading causes of death for this group of patients. The older the patient, the lower the male:female ratio becomes (Cooper and Stroehla 2003).

Although the etiology of ADs is unknown, these diseases are known to feature genetic and environmental factors in their development (Anaya et al. 2005; Vyse and Todd 1996). The impact of genetic predisposition on susceptibility to ADs was first identified by the analysis of disease concordance rates in monozygotic twins. The monozygotic disease concordance rate ranges from about 15% for rheumatoid arthritis (RA) (Silman et al. 1993) to a fairly robust 57% for systemic lupus erythematosus (SLE) (Winchester 1992) climate. Comparisons of these high concordance rates with disease incidence in the general population predict that genetic predisposition is the dominant factor in AD susceptibility. The dramatic decrease in the concordance rate of

Correspondence: J.-M. Anaya, Cellular Biology and Immunogenetics Unit, Corporación para Investigaciones Biológicas, Cra. 72-A No 78-B-141, Medellín, Colombia. Tel: 574 441-08-55. Fax: 574 441-55-14. E-mail: janaya@cib.org.co

ISSN 1740-2522 print/ISSN 1740-2530 online © 2006 Taylor & Francis
DOI: 10.1080/17402520600876762

siblings compared with that of monozygotic twins supports the presence of multiple genes contributing to the genetic predisposition. Finally, the estimation of familial aggregation, or recurrent risk ratio ($\lambda_R$) for ADs (which is the ratio of the risk of disease prevalence among the specific-relative pairs ($_R$) of affected probands to the prevalence of the disease in the general population), supports a fundamental role for genetic predisposition in disease susceptibility (Anaya et al. 2005). However, population studies have established that each population holds a mutational pool, in which most mutations (i.e. polymorphisms) have mild or even undetectable effects individually, but in combination with other alleles may promote or protect from, autoimmune phenomena. Such interplay between genetic variants will generate a change in the measurable risk of developing an autoimmune phenotype. This characteristic is the main reason why ADs are not inherited in a simple, classical Mendelian way, but instead have a complex or an as yet unknown mode of inheritance (Vyse and Todd 1996).

As a group, ADs represent a diverse collection of diseases in terms of their demographic profile and primary clinical manifestations. The commonality between them, however, is the damage to tissues and organs that arises from the response to self-antigens (loss of tolerance). The vast majority of research generally focuses strongly on individual clinical diseases even though these autoimmune phenotypes could represent pleiotropic outcomes of specific, common disease genes that underlie similar immunogenetic mechanisms (Anaya et al. 2005). Therefore, our group is investigating in a well-defined population whether or not clinically different ADs share the same susceptibility risk factors (i.e. genetic variants). This hypothesis of a common genetic origin for diverse ADs has been sustained by two important findings. The first consisted of a clinical analysis in which aggregation of ADs was observed in families of patients with primary Sjögren's syndrome (pSS) and type 1 diabetes mellitus (T1D). The second was a large genetic association study carried out in three different ADs namely RA, SLE and pSS. Herein, we summarize the main results of these and other similar studies that reveal new insights concerning the inheritance pattern and the genetic risk factors for the development of ADs.

### Genetic history of the sample population

Historical and genetic evidence suggests that the population of Antioquia is useful for the genetic mapping of complex traits. The state of Antioquia (capital, Medellín) is geographically located in the north-western part of Colombia between the Central and Western branches of the Andean Mountains and is inhabited by the "Paisa" community, a description of which has already been published (Bravo et al. 1996; Jimenez et al. 1996). Anthropological and historical studies describe this population as the most clearly defined in Colombia. Its ethno-historical origin stems most probably from the Spaniards, Jews (Christianized Sephardim or Marranos), and Basques. The admixture between Paisa and African or Amerindian populations has been historically documented as low (Parsons 1968). Several lines of genetic evidence suggest that the Paisa community exhibits the features of a genetically isolated community. Firstly, the identity coefficient method has estimated the ancestral ethnic components as 85% Caucasian and 15% Amerindian (Bravo et al. 1996). The African contribution to the Paisa community was estimated as being not significantly different from 0 (Jimenez et al. 1996). Secondly, strong admixture distortions in the gender vectors of racial blending in this community were found, with more than 96% of the chromosomes being of Caucasian origin and most of the mitochondrial component of Amerindian origin (Carvajal-Carmona et al. 2000).

### Familial aggregation of autoimmunity

Primary SS is a late-onset AD characterized by a lymphocytic and plasma cell infiltration of the exocrine glands, as well as by the production of autoantibodies leading to dryness of mucosa, mainly oral and lachrymal (Anaya et al. 2001). Contrary to pSS, T1D appears early in life as a consequence of autoimmune damage to the insulin-producing pancreatic β cells (Expert Committee on the Diagnosis and Classification of Diabetes Mellitus 1997). There is strong evidence indicating familial clustering of various ADs in patients with several ADs including pSS and T1D (Anaya et al. 2006a, c) (Table I).

Bloch and Bunin first suggested a shared immunopathological mechanism for SS, SLE, systemic sclerosis (SSc) and autoimmune thyroid diseases (AITD), as well as a possible familial aggregation of these diseases in pSS patients (Bloch and Bunim 1963). They described the clinical and immunological characteristics of 57 cases of SS and some immunological abnormalities among their relatives. However, most of those patients (70%) had secondary SS (Bloch and Bunim 1963). In fact, SS may coexist with other ADs such as RA (Moutsopoulos et al. 1979), SLE (Steinberg and Talal 1971), AITD (Foster et al. 1993), and SSc (Alarcon-Segovia et al. 1974). Later, Reveille et al (1984) examined the presence of ADs in family members of 51 patients with pSS. They investigated the relationships of *HLA* genes and heavy chain immunoglobulin (Gm) haplotypes to disease and autoantibody expression in six large kindreds, each having one or more members with pSS.

Table I. Main familial autoimmunity reports.

| PD | No. of families | Type of study | Aggregated disease | Population | Observations | References |
|---|---|---|---|---|---|---|
| ADs | 265 | Multiplex families | T1D, RA, SLE | MADGC | AD Core: RA, SLE, T1D, MS, AITD, JRA, IBD, PSO, pSS | Criswell et al. (2005) |
| ADs | | Review analysis | MS with AITD, ADD, SLE, pSS | | AITD co-ocurrence | Sloka (2002) |
| JRA | 164 | Affected-Sibbling pairs | AITD | USA | | Moroldo et al. (2004) |
| JRA | 110 cases 45 control | Case-control family | AITD | USA | Autoimmunity prevalence higher in FDR than in SDR | Prahalad et al. (2002) |
| MS | 571 | Case-control family study | AITD | Caucasian-UK | $\lambda_S = 1.65$ | Broadley et al. (2000) |
| MS | 357 | Multiplex families | MS, T1D, AITD, VIT | French | | Heinzlef et al. (2000) |
| pSS | 2 patients | Case report | Connective tissue diseases | Japanese | HLA haplotypes do not explain alone autoimmunity | Moriuchi et al. (1986) |
| pSS | Twin patients and their mother | Case report | pSS and Immunological diseases | | All pSS, similar clinical, serological data and histological data. History of immunological disorders | Bolstad et al. (2000) |
| pSS | 98 | Multiplex families | AITD, SLE, pSS, MS, SSc | | Significant sex effect | Reveille et al. (1984) |
| pSS | 101 patients 124 controls | Multiplex families | AITD, SLE, RA | Colombian | SSc and AITD correlated with proband's phenotype | Anaya et al. (2006c) |
| RA | 11 patients | Gene expression array profiles on FDR | SLE, RA, MS, T1D | USA | Transcript levels are associated with family resemblance | Maas et al. (2005) |
| RA | 257 patients | Genomewide screen in multiplex families | RA, SLE, IBD, AS | USA | Allele sharing for HLA and other regions | Jawaheer et al. (2001) |
| SLE | 11 patients | Gene expression array profiles on FDR | SLE, RA, MS, T1D | USA | Transcript levels are associated with family resemblance | Maas et al. (2005) |
| SLE | 154 cases 140 controls | Multicentric retrospective case-control study | SLE | Italian | SLE FDR susceptibility risk increased | Priori et al. (2003) |
| SLE | 118 patients | Multicase SLE families | SLE, MS, RA | Caucasian | Higher prevalence of AD in relatives of Probands | Corporaal et al. (2002) |
| SLE | 12 twin pairs | Case report | | | $\lambda_{MZ} = 57$; DZ were discordant for SLE | Block et al. (1975) |
| SLE | 1214 patients | Multiplex families | RA | GLADEL | $\lambda_S = 1.5$ | Alarcon-Segovia et al. (2005) |
| T1D | 505 families | Affected-sibling families | T1D | UK | Parents AD prevalence higher compared to the population | Tait et al. (2004) |
| TID | 98 patients 113 controls | Multiplex families | AITD, T1D | Colombian | $\lambda_S = 2.21$ ADs taken together | Anaya et al. (2006a) |
| VIT | 2624 patients | Multiplex families | VIT, AITD, PA, ADD, SLE | USA-UK Caucasian | $\lambda_S = 6.1$ | Alkhateeb et al. (2003) |
| VIT | 133 patients | Multiplex families | AITD, RA, PSO, T2D, ADD, PA | Caucasian-UK and USA | $\lambda_S = 6.34$ | Laberge et al. (2005) |

PD, proband's disease; MADGC, Multiple Autoimmune Disease Genetics Consortium; GLADEL, Grupo Latinoamericano de Estudio del Lupus Eritematoso Sistémico; AD, autoimmune disease; pSS, primary Sjogren's syndrome; T1D, type 1 diabetes mellitus; AITD, autoimmune thyroid diseases; SLE, systemic lupus erythematosus; RA, rheumatoid arthritis; SSc, systemic sclerosis; PBC, primary biliary cirrhosis; VIT, vitiligo; MS, multiple sclerosis; PSO, psoriasis; T2D, type 2 diabetes; ADD, Addison's disease; IBD, inflammatory bowel disease.

Segregation analyses suggested a Mendelian dominant genetic effect common among the many ADs and serologic reactions that were not linked to HLA or Gm (Reveille et al. 1984). Recently, we performed a familial aggregation study of ADs in 101 families of pSS patients and 124 control families (Anaya et al. 2006c). In the families of pSS patients, 38 (37.6%) had at least one first degree relative (FDR) with an AD, compared with 27 (21.8%) of control families (OR 2.2, 95% CI: 1.2–3.9, $p = 0.01$). Similarly to Reveille et al, we found that AITD, RA, and SLE were the most common ADs among relatives of the pSS patients (Reveille et al. 1984; Anaya et al. 2006c). Multiple sclerosis and SSc were also observed, although with lower frequency. Moreover, the risk of familial autoimmunity increased with the number of affected FDR (Anaya et al. 2006c). Familial aggregation of AITD, SLE, RA, T1D, vitiligo (VIT), and all ADs taken together as a single trait, was observed (Anaya et al. 2006c).

Familial autoimmunity was also investigated in 98 T1D families and 113 families of healthy control individuals all from the same Colombian population (Anaya et al. 2006a). Our results showed that in the families of T1D patients, 25 (25.5%) presented at least one FDR having an AD compared with 9 (8%) in control families (OR: 3.96, 95% CI: 1.75–9; $p = 0.0006$), supporting the findings of Tait et al (2004) who observed a similar increase of ADs in family members of British patients with T1D. In our cohort, familial aggregation was observed for AITD, T1D and all ADs taken together as a single trait (Anaya et al. 2006a). Of particular interest, AITD (mainly hypothyroidism) is the most common AD encountered among FDR of pSS and T1D patients; this has also been reported in familial studies in multiple sclerosis (Broadley et al. 2000), VIT (Alkhateeb et al. 2003), juvenile RA (JRA) (Prahalad et al. 2002), and SLE (Priori et al. 2003). The next most common diagnosed ADs in FDR of pSS patients are RA and SLE, while in T1D SLE fills this position (Anaya et al. 2006a, c). All these diseases, as we will discuss, share similar susceptibility gene polymorphisms including HLA and non-HLA variants (Becker et al. 1998; Wandstrat and Wakeland 2001; Correa et al. 2005; Gomez et al. 2005), which may account for aggregation. In fact, shared genetic factors are the most likely cause of familial aggregation; however, it is important to keep in mind that shared environmental factors can also contribute to such aggregation. For a specified relative type, a $\lambda_R > 1.0$ suggests familial aggregation of the disease, but does not identify whether genetic or environmental factors are aggregating (Laird and Cuenco 2003). Thus, familial aggregation studies should be control studies in which population matched proband families lacking the trait of interest should be included.

*Maternal transmission of the autoimmunity trait*

A predominant inheritance of the autoimmunity trait from mothers has been observed in patients with pSS (Reveille et al. 1984; Anaya et al. 2006c), SLE (Priori et al. 2003) and T1D (Tait et al. 2004), indicating a plausible and preferential transmission of susceptibility alleles from mothers to offspring. Maternal transmission of autoimmunity could be influenced by the high preponderance of ADs in females compared to the general population. However, this higher than expected frequency of the autoimmunity trait maternal transmission would warrant further studies of mtDNA, genomic imprinting, maternal-offspring compatibility, and indirect genetic effects.

Collectively, results indicate that in pSS, T1D as well as in other ADs, pathologic autoimmunity aggregates as a trait (Table I), and emphasize the importance of the autoimmunity family history as a substantial risk factor for the development of any AD. This summary also points out the magnitude of the autoimmunity family history when carrying out both linkage and association genetic studies (i.e. transmission disequilibrium test).

### Population genetic evidence for the common origin of ADs

The autoimmune phenotype and genotype might vary among populations. Diverse populations would behave genetically different when referring to its genetic variants, depending on the population natural and epidemiological history (Mori et al. 2005). In addition, the effects of genotype on phenotype in any given population may depend upon environment and the length of exposure to an undefined etiological insult. Therefore, there is a need to explore genetic associations in diverse populations. Confirming the results within different populations favors a more complete and homogeneous comprehension of the pathogenic mechanisms of ADs.

The "common variants/multiple disease (CV/MD)" hypothesis implies that "complex phenotypes are not unique entities but are mosaics of common disease specific alleles and non-disease specific modifying alleles in the population, influenced by a vast array of environmental factors" (Becker 2004). We surmise that if the CV/MD hypothesis was to be validated in an autoimmune setting, it would provide a convincing framework for the possibility that a common set of alleles might contribute to dissimilar clinical phenotypes.

In order to further investigate the common origin for diverse ADs we examined the polymorphisms of 10 genes in different ADs in a Northwestern Colombian population. These genes were chosen by their important participation in autoimmune response and inflammation (Table II). Our results demonstrated

Table II. Genetic polymorphisms associated with ADs in Northwestern Colombians.

| Gene | Location | AD | Associated variant | OR (95% CI) | *p*-value | References |
|---|---|---|---|---|---|---|
| *HLA-DRB1* | 6p21.3 | RA | *HLA-DRB1**0404 | 3.7 (1.73–7.83) | 0.0009 | Anaya et al. (2002a) |
| *HLA-DQB1* | 6p21.3 | pSS | *HLA-DRB1**0301-DQB1*0201 | 4.3 (1.6–11.9) | 0.001 | Anaya et al. (2002b) |
| | | SLE | *HLA-DRB1**0301-DQB1*0201 | 3.12 (1.45–6.73) | 0.003 | Anaya et al. (2006b) |
| *TAP2* | 6p21.3 | SLE | *TAP2**0201 | 2.0 (1.22–3.30) | 0.03 | Anaya et al. (2002b) |
| | | RA | *TAP2**0201 | 3.0 (1.5–5.6) | 0.002 | Anaya and Correa (2000) |
| *TNF* | 6p21.3 | RA | *TNF*-308A | 1.8 (1.26–2.54) | 0.002 | Correa et al. (2005) |
| | | SLE | *TNF*-308A | 2.6 (1.77–3.83) | 0.0001 | |
| | | pSS | *TNF*-308A | 2.9 (1.90–4.57) | 0.0001 | |
| *NFKBIL1* | 6p21.3 | SLE | *IKBL* + 738T | 0.45 (0.24–0.83) | 0.016 | Castiblanco and Anaya (2006) |
| | | pSS | *IKBL* + 738T | 0.38 (0.18–0.74) | 0.008 | |
| | | SLE | *IKBL*-62A | 0.59 (0.43–0.79) | 0.0004 | |
| *NOS3* | 7q36.1 | SLE | Intron 4b | 2.2 (1.29–3.60) | 0.005 | Serrano et al. (2004) |
| *PTPN22* | 1p13.2 | SLE | 1858T | 2.58 (1.49–4.39) | 0.001 | Gomez et al. (2005) |
| | | pSS | 1858T | 2.42 (1.24–4.75) | 0.01 | |
| | | T1D | 1858T | 1.83 (0.98–3.42) | 0.06 | |
| *CCR5* | 3p21.31 | SLE | HHE haplotype | 1.98 (1.12–3.53) | 0.001 | Herrera et al. (2004) |
| | | | HHG*2 haplotype | 2.55 (1.69–3.85) | 0.001 | |
| *IL1B* | 2q13 | SLE | +3953T | 0.57 (0.34–0.88) | 0.01 | Camargo et al. (2004) |

RA, rheumatoid arthritis; SS, Sjögren's syndrome; SLE, systemic lupus erythematosus; T1D, Type 1 diabetes mellitus.

that the *HLA-DRB1* gene, *TAP2**0201, *TNF2*, and *PTPN22* 1858T alleles significantly influence the susceptibility for acquiring diverse ADs, while variants of *CCR5* and *NOS3* genes influence the development of a particular AD (such as SLE: Table II). Furthermore, we have found that *NFKBIL1* gene polymorphisms behave as protective factors for the development of SLE and pSS, while *IL1B* polymorphism protect against the development of SLE only (Table II). Taken together, our results clearly indicate that in a single, carefully-characterized population, some polymorphisms are common risk factors for the development of ADs while others are disease specific (Figure 1).

Independent reverse genetic studies have also shown that other polymorphic genes could influence the susceptibility to acquire multiple ADs (i.e. *CTLA4, CARD15, FCGR2A, FCRL3, IFNG, NOS2A, PARP1, PDCD1, RUNX1, MIF*) (Oertelt et al. 2005; Yamada and Ymamoto 2005; Pearce and Merriman 2006; Serrano et al. 2006). Findings from the Multiple Autoimmune Disease Genetics Consortium (MADGC) have also shown that clinically distinct autoimmune phenotypes might share a common set of susceptibility genes (Criswell et al. 2005). Likewise, results from linkage studies in different populations have shown analogous results, which are summarized in Table III.

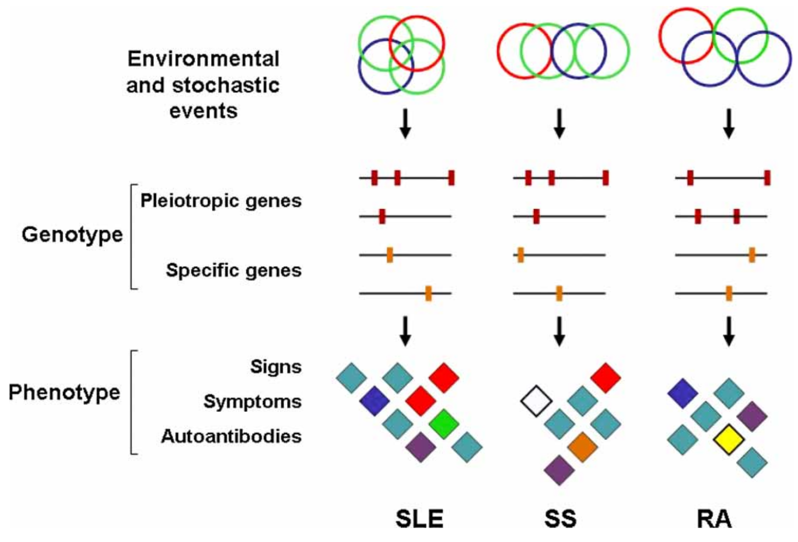

Figure 1. The common origin for diverse ADs hypothesis. ADs are the result of multiple interactions of polymorphic genes and environmental or stochastic factors leading to a loss of immunological tolerance to self-antigens and then to tissue damage. The genetic risk factors for ADs may well consist of two forms: those which are common to many ADs acting in pleitropy, and those that are specific to a given disorder. SLE: systemic lupus erythematosus, SS: Sjögren's syndrome, RA: rheumatoid arthritis.

Table III. Chromosomal regions associated or linked with autoimmune traits.

| | AD | | | | | |
|---|---|---|---|---|---|---|
| Chr. | SLE | RA | AITD | MS | T1D | IBD |
| 1 | 1q41 (Graham et al. 2001) | 1p13, 1q43 (Jawaheer et al. 2003) | | 1p21.3 (Becker et al. 1998) | 1q42 (Cox et al. 2001) | |
| | 1p36, 1p13, 1q42 (Gaffney et al. 1998) | | | 1p34 (Pericak-Vance et al. 2004) | | |
| | 1q23 (Tsao et al. 2002) | | | | | |
| 2 | 2q37 (Lindqvist et al. 2000) | | | 2p11 (D'Alfonso et al. 1999) | 2q31-34 (Myerscough et al. 2000) | 2q32.3 (Duerr et al. 2000) |
| | 2p15, 2q21-33 (Gaffney et al. 1998) | | | | 2q31 (Cox et al. 2001) | |
| 3 | 3cent-q11 (Gaffney et al. 1998) | | | 3p21.2, 3p14.1, 3p13 (Becker et al. 1998) | | 3q13.12 (Duerr et al. 2000) |
| | | | | 3p14 (Pericak-Vance et al. 2004) | | |
| | | | | 3q21.1 (D'Alfonso et al. 1999) | | |
| 4 | 4q28 (Gaffney et al. 1998) | | | | | |
| | 4q15-13 (Lindqvist et al. 2000) | | | | | |
| 5 | 5q14.3-15 (Namjou et al. 2005) | 5q14.3-15 (Namjou et al. 2005) | | 5p14-12 (Kuokkanen et al. 1997) | | 5q33-q35 (Ma et al. 1999) |
| | | | | 5p14 (Dyment et al. 2001) | | |
| | | | | 5p12-13 (Mellai et al. 2003) | | |
| | | | | 5p (Oturai et al. 1999) | | |
| 6 | 6q (Koskenmies et al. 2004) | 6q (Eyre et al. 2004) | 6p (Tomer et al. 2003) | 6p (Mellai et al. 2003) | 6q27 6p21 (Cox et al. 2001) | |
| | 6p11-21 (Gaffney et al. 1998) | 6p21.3 (Jawaheer et al. 2003) | 6p22.3-14.1 (Alkhateeb et al. 2002) | 6p21 (Kuokkanen et al. 1997) | 6q25-27 (Myerscough et al. 2000) | |
| | | 6p21.3 (Dyment et al. 2001) | | | 6p21 (Oturai et al. 1999) | |
| | | 6p21, 6q (Fisher et al. 2003) | | | | |
| 7 | | | 7q (Tomer et al. 2003) | 7p15.2, 7q11.21 (Becker et al. 1998) | 7p15.2, 7q31.31 (Becker et al. 1998) | 7p15.2 (Becker et al. 1998) |
| | | | | 7p15.2 (D'Alfonso et al. 1999) | | 7q32.1 (Duerr et al. 2000) |
| 8 | | | 8q (Tomer et al. 2003) | | 8q24 (Sale et al. 2002) | |
| 9 | 9p22 (Lindqvist et al. 2000) | 9p22 (Jawaheer et al. 2003) | | | | |
| 10 | | 10q21 (Jawaheer et al. 2003) | 10q (Tomer et al. 2003) | 10q26.3 (Becker et al. 1998) | 10q26.3 (Becker et al. 1998) | |
| | | | | | 10p11 (Cox et al. 2001) | |
| 11 | 11p15 (Gaffney et al. 1998) | | | 11p15.5 (Becker et al. 1998) | 11p15.5 (Becker et al. 1998) | |
| | | | | | 11p15 (Cox et al. 2001) | |
| 12 | | 12q12 (Jawaheer et al. 2003) | | 12p13.12 (Becker et al. 1998) | 12p13.12 (Becker et al. 1998) | 12q (Duerr et al. 2000) |
| | | 12p (Fisher et al. 2003) | | | | |
| 13 | | | | 13q (Becker et al. 1998) | 13q (Becker et al. 1998) | |

Table III – *Continued*

| Chr. | AD | | | | | |
|---|---|---|---|---|---|---|
| | SLE | RA | AITD | MS | T1D | IBD |
| 14 | 14q (Koskenmies et al. 2004) | | 14q (Tomer et al. 2003) | | | 14q11-12 (Duerr et al. 2000) |
| | 14q21-23 (Gaffney et al. 1998) | | | | | 14q11.2 (Ma et al. 1999) |
| 15 | 15q26 (Gaffney et al. 1998) | | | | | |
| 16 | 16q22 (Tsao et al. 2002) | 16cen (Fisher et al. 2003) | | | 16q22 (Cox et al. 2001) | 16q12.1 (Duerr et al. 2000) |
| | 16q13 (Gaffney et al. 1998) | 16p (Eyre et al. 2004) | | | | |
| 17 | | 17q13 (Jawaheer et al. 2003) | | 17p13.3 (Becker et al. 1998) | 17p13.3 (Becker et al. 1998) | |
| | | | | 17q (Larsen et al. 2000) | | |
| | | | | 17q22-24 (Kuokkanen et al. 1997) | | |
| | | | | 17q12 (Dyment et al. 2001) | | |
| 18 | | 18q21 (Jawaheer et al. 2003) | 18q21 (Vaidya et al. 2000) | | | 18q22.2 (Duerr et al. 2000) |
| 19 | 19q13 (Becker et al. 1998) | | | 19q13 (Becker et al. 1998) | | |
| | 19q13 (Lindqvist et al. 2000) | | | 19q13 (Pericak-Vance et al. 2004) | | |
| 20 | 20p12 (Gaffney et al. 1998) | | 20q (Tomer et al. 2003) | | | |
| 21 | | | | | | |
| 22 | | | | 22q13.1 (D'Alfonso et al. 1999) | | |
| X | | | | Xp11.1 (Becker et al. 1998) | Xp11.1 (Becker et al. 1998) | |

MS, multiple sclerosis; SLE, systemic lupus erythematosus; T1D, type 1 diabetes; RA, rheumatoid arthritis; AITD, autoimmune thyroid diseases; IBD, inflammatory bowel disease.

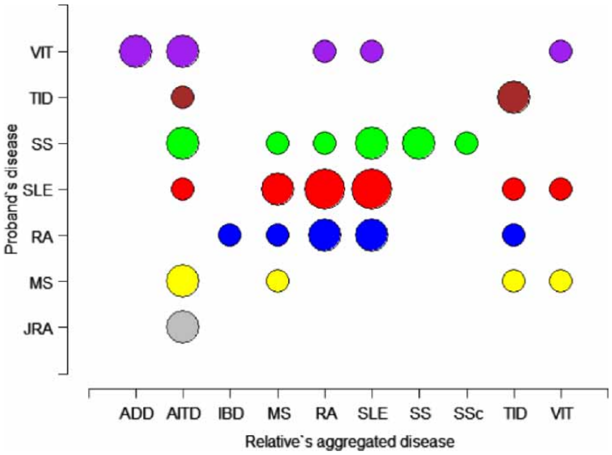

Figure 2. Familial autoimmunity clusters. The ordinate represents the proband's disease, while the axis despites the correspondent aggregated AD. Circle size represents the weighted analyzed reports and does not necessarily indicate the aggregation's magnitude. This figure was generated from Table I data. JRA, juvenile rheumatoid arthritis; MS, multiple sclerosis; SS, Sjögren's syndrome; RA, rheumatoid arthritis; SLE, systemic lupus erythematosus; T1D, type 1 diabetes mellitus; VIT, vitiligo; AITD, autoimmune thyroid diseases; ADD, Addison's disease; IBD, inflammatory bowel disease; SSc, systemic sclerosis.

The aforementioned findings indicate that ADs might be the consequence of pleiotropic effects of specific genes on a common polygenic background (Reveille et al. 1984; Bias et al. 1986; Lin et al. 1998). Strong suggestions from previous studies allow us to point out the major histocompatibility complex, including both HLA and non-HLA loci, as one of the central loci contributing to pSS, T1D and other ADs (Vyse and Todd 1996; Wandstrat and Wakeland 2001). However, not all ADs share the same genetic susceptibility or allelic spectrum. Thus, the genetic risk factors for ADs may well consist of two forms: those which are common to many ADs and those that are specific to a given disorder (Figure 1).

### Conclusion and perspectives

Herein, we have uncovered two approaches supporting the hypothesis of a common genetic origin for diverse ADs. A familial core for ADs depending upon the proband phenotype is illustrated as a graphic contingency table in Figure 2, and the chromosomal regions overlapping among the most frequent ADs are depicted in Figure 3. The precise mechanisms by which possession of the genetic variants affect diverse ADs are not completely understood and are beyond the scope of this review. However, genetic variation plays an important role in the determination of individual changes in protein expression as discussed elsewhere (Price et al. 1999; Camargo et al. 2004; Knight 2005; Castiblanco and Anaya 2006; Serrano et al. 2006). Since variation in gene expression is heritable and can be mapped as a quantitative trait, haplotype structures and functional assays should be considered in such genetic studies.

The co-occurrence of ADs is a well-known phenomenon. Epidemiological studies have described an increased statistical susceptibility of people with one AD to develop other ADs (Sloka 2002). This

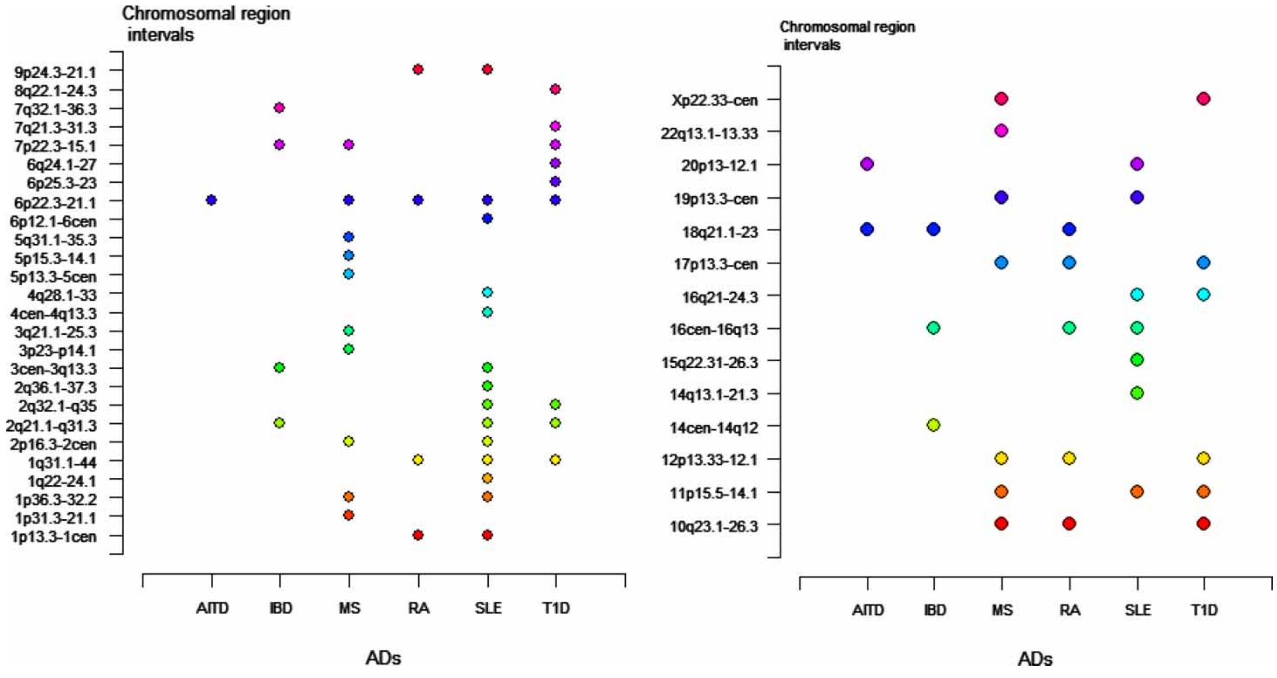

Figure 3. Chromosome regions overlap among ADs. The ordinate represents the AD, while the axis despites the chromosomal region intervals. This figure was generated from table III data. AITD: autoimmune thyroid diseases, IBD, inflammatory bowel disease; MS, multiple sclerosis; RA, rheumatoid arthritis; SLE, systemic lupus erythematosus; T1D, type 1 diabetes mellitus.

situation is well illustrated by the type 2 autoimmune polyglandular syndrome, originally described by Schmidt (1926), and by the "multiple autoimmune syndrome", described by Humbert and Dupond (1988). In these scenarios a single genotype is responsible for diverse phenotypes, making these cases the "magna prove" for a common origin of ADs. Recently, Namjou et al. (2005) after stratification of multiplex SLE pedigrees by the presence of AITD, observed that 5q14.3–15 is a region linked to both diseases. Thus, besides autoimmunity family history, the co-occurrence of ADs should be also considered when performing association and linkage studies.

The common genetic background evidence for ADs has grown and allows us to infer that AD phenotypes have a clinical behaviour that can be independent from their genetic causes. The heterogeneity of ADs could be due to a collection of diverse disorders based on epidemiology pathology or diagnostic results but in fact the underlying immunogenetic mechanism might be similar. Identification of such common genetic causes will enhance our understanding of the common mechanisms of these complex, frequent and sometimes devastating diseases and will permit us to predict them as well as to discover new therapeutic interventions.

### Acknowledgements

We thank all the patients and participants of our study as well as our colleagues Grant Gallagher, Sunil Ahuja, Javier Martin, Norma Serrano, Yehuda Shoenfeld, Paula Correa, Ricardo Pineda-Tamayo, Gabriel J. Tobón, Jose F. Camargo, Jose Cadena and Ruben D. Mantilla for their assistance and advice. We apologize for our inability to reference several additional excellent articles on this subject because of space constraints. This project has been financed partially by Colciencias (Bogotá) and Fundación Social TCC (Medellín). We dedicate this work to Angela Restrepo and William Rojas, for their constant support and mentoring.

### References

Alarcon-Segovia D, Alarcon-Riquelme ME, Cardiel MH, Caeiro F, Massardo L, Villa AR, Pons-Estel BA. 2005. Familial aggregation of systemic lupus erythematosus, rheumatoid arthritis, and other autoimmune diseases in 1,177 lupus patients from the GLADEL cohort. Arthritis Rheum 52:1138–1147.

Alarcon-Segovia D, Ibanez G, Hernandez-Ortiz J, Velazquez-Forero F, Gonzalez-Jimenez Y. 1974. Sjogren's syndrome in progressive systemic sclerosis (scleroderma). Am J Med 57:78–85.

Alkhateeb A, Fain PR, Thody A, Bennett DC, Spritz RA. 2003. Epidemiology of vitiligo and associated autoimmune diseases in Caucasian probands and their families. Pigment Cell Res 16:208–214.

Alkhateeb A, Stetler GL, Old W, Talbert J, Uhlhorn C, Taylor M, Fox A, Miller C, Dills DG, Ridgway EC, et al. 2002. Mapping of an autoimmunity susceptibility locus (AIS1) to chromosome 1p31.3–p32.2. Hum Mol Genet 11:661–667.

Anaya JM, Castiblanco J, Tobon GJ, Garcia J, Abad V, Cuervo H, Velasquez A, Angel ID, Vega P, Arango A. 2006a. Familial clustering of autoimmune diseases in patients with type 1 diabetes mellitus. J Autoimmun, 67:290.

Anaya JM, Correa PA. 2000. Análisis molecular de los alelos HLA-DRB1 y TAP2 asociados a la susceptibilidad de la arthritis reumatoidea. Acta Med Colomb 25:290.

Anaya JM, Correa PA, Mantilla RD, Arcos-Burgos M. 2002a. Rheumatoid arthritis association in Colombian population is restricted to HLA-DRB1*04 QRRAA alleles. Genes Immun 3:56–58.

Anaya JM, Correa PA, Mantilla RD, Arcos-Burgos M. 2002b. TAP, HLA-DQB1, and HLA-DRB1 polymorphism in Colombian patients with primary Sjogren's syndrome. Semin Arthritis Rheum 31:396–405.

Anaya JM, Pineda-Tamayo R, Rojas-Villaraga A. 2006b. HLA-DRB1 and DQB1 polymorphism in Northwester Colombian patients with systemic lupus erythematosus. Clin Rheumatol, submitted.

Anaya JM, Ramos M, García M. 2001. Síndrome de Sjögren. Medellin: Corporación para Investigaciones Biológicas.

Anaya JM, Shoenfeld Y, Correa PA, Garcia-Carrasco M, Cervera R. 2005. Autoimmunity and autoimmune disease. 1 ed. Medellin: CIB.

Anaya JM, Tobon GJ, Vega P, Castiblanco J. 2006c. Autoimmune disease aggregation in primary Sjögren's syndrome families. J Rheumatol, in press.

Becker KG. 2004. The common variants/multiple disease hypothesis of common complex genetic disorders. Med Hypotheses 62:309–317.

Becker KG, Simon RM, Bailey-Wilson JE, Freidlin B, Biddison WE, McFarland HF, Trent JM. 1998. Clustering of non-major histocompatibility complex susceptibility candidate loci in human autoimmune diseases. Proc Natl Acad Sci USA 95:9979–9984.

Bias WB, Reveille JD, Beaty TH, Meyers DA, Arnett FC. 1986. Evidence that autoimmunity in man is a Mendelian dominant trait. Am J Hum Genet 39:584–602.

Block SR, Winfield JB, Lockshin MD, D'Angelo WA, Christian CL. 1975. Studies of twins with systemic lupus erythematosus. A review of the literature and presentation of 12 additional sets. Am J Med 59:533–552.

Bloch KJ, Bunim JJ. 1963. Sjögren's syndrome and its relation to connective tissue diseases. J Chronic Dis 16:915–927.

Bolstad AI, Haga HJ, Wassmuth R, Jonsson R. 2000. Monozygotic twins with primary Sjogren's syndrome. J Rheumatol 27:2264–2266.

Bravo ML, Valenzuela CY, Arcos-Burgos OM. 1996. Polymorphisms and phyletic relationships of the Paisa community from Antioquia [Colombia]. Gene Geogr 10:11–17.

Broadley SA, Deans J, Sawcer SJ, Clayton D, Compston DA. 2000. Autoimmune disease in first-degree relatives of patients with multiple sclerosis. A UK survey. Brain 123((Pt 6)):1102–1111.

Camargo JF, Correa PA, Castiblanco J, Anaya JM. 2004. Interleukin-1beta polymorphisms in Colombian patients with autoimmune rheumatic diseases. Genes Immun 5:609–614.

Carvajal-Carmona LG, Soto ID, Pineda N, Ortiz-Barrientos D, Duque C, Ospina-Duque J, McCarthy M, Montoya P, Alvarez VM, Bedoya G, Ruiz-Linares A. 2000. Strong Amerind/white sex bias and a possible Sephardic contribution among the founders of a population in northwest Colombia. Am J Hum Genet 67:1287–1295.

Castiblanco J, Anaya JM. 2006. Role of the NFKBIL1 within the class III MHC and influence in autoimmune rheumatological diseases. J Autoimmun, submitted.

Cooper GS, Stroehla BC. 2003. The epidemiology of autoimmune diseases. Autoimmun Rev 2:119–125.

Corporaal S, Bijl M, Kallenberg CG. 2002. Familial occurrence of autoimmune diseases and autoantibodies in a Caucasian population of patients with systemic lupus erythematosus. Clin Rheumatol 21:108–113.

Correa PA, Gomez LM, Cadena J, Anaya JM. 2005. Autoimmunity and tuberculosis. Opposite association with TNF polymorphism. J Rheumatol 32:219–224.

Cox NJ, Wapelhorst B, Morrison VA, Johnson L, Pinchuk L, Spielman RS, Todd JA, Concannon P. 2001. Seven regions of the genome show evidence of linkage to type 1 diabetes in a consensus analysis of 767 multiplex families. Am J Hum Genet 69:820–830.

Criswell LA, Pfeiffer KA, Lum RF, Gonzales B, Novitzke J, Kern M, Moser KL, Begovich AB, Carlton VE, Li W, et al. 2005. Analysis of families in the multiple autoimmune disease genetics consortium (MADGC) collection: the PTPN22 620W allele associates with multiple autoimmune phenotypes. Am J Hum Genet 76:561–571.

D'Alfonso S, Nistico L, Zavattari P, Marrosu MG, Murru R, Lai M, Massacesi L, Ballerini C, Gestri D, Salvetti M, et al. 1999. Linkage analysis of multiple sclerosis with candidate region markers in Sardinian and Continental Italian families. Eur J Hum Genet 7:377–385.

Duerr RH, Barmada MM, Zhang L, Pfutzer R, Weeks DE. 2000. High-density genome scan in Crohn disease shows confirmed linkage to chromosome 14q11–12. Am J Hum Genet 66:1857–1862.

Dyment DA, Willer CJ, Scott B, Armstrong H, Ligers A, Hillert J, Paty DW, Hashimoto S, Devonshire V, Hooge J, et al. 2001. Genetic susceptibility to MS: a second stage analysis in Canadian MS families. Neurogenetics 3:145–151.

Expert Committee on the Diagnosis and Classification of Diabetes Mellitus 1997. Expert Committee on the Diagnosis and Classification of Diabetes Mellitus Report of the expert committee on the diagnosis and classification of diabetes mellitus. Diabetes Care 26(Suppl 1):S5–20.

Eyre S, Barton A, Shephard N, Hinks A, Brintnell W, MacKay K, Silman A, Ollier W, Wordsworth P, John S, Worthington J. 2004. Investigation of susceptibility loci identified in the UK rheumatoid arthritis whole-genome scan in a further series of 217 UK affected sibling pairs. Arthritis Rheum 50:729–735.

Fisher SA, Lanchbury JS, Lewis CM. 2003. Meta-analysis of four rheumatoid arthritis genome-wide linkage studies: confirmation of a susceptibility locus on chromosome 16. Arthritis Rheum 48:1200–1206.

Foster H, Fay A, Kelly C, Charles P, Walker D, Griffiths I. 1993. Thyroid disease and other autoimmune phenomena in a family study of primary Sjogren's syndrome. Br J Rheumatol 32:36–40.

Gaffney PM, Kearns GM, Shark KB, Ortmann WA, Selby SA, Malmgren ML, Rohlf KE, Ockenden TC, Messner RP, King RA, et al. 1998. A genome-wide search for susceptibility genes in human systemic lupus erythematosus sib-pair families. Proc Natl Acad Sci U S A 95:14875–14879.

Gomez LM, Anaya JM, Gonzalez CI, Pineda-Tamayo R, Otero W, Arango A, Martin J. 2005. PTPN22 C1858T polymorphism in Colombian patients with autoimmune diseases. Genes Immun 6:628–631.

Graham RR, Langefeld CD, Gaffney PM, Ortmann WA, Selby SA, Baechler EC, Shark KB, Ockenden TC, Rohlf KE, Moser KL, et al. 2001. Genetic linkage and transmission disequilibrium of marker haplotypes at chromosome 1q41 in human systemic lupus erythematosus. Arthritis Res 3:299–305.

Heinzlef O, Alamowitch S, Sazdovitch V, Chillet P, Joutel A, Tournier-Lasserve E, Roullet E. 2000. Autoimmune diseases in families of French patients with multiple sclerosis. Acta Neurol Scand 101:36–40.

Herrera M, Gonzalez E, Kulkarni H, Correa PA, Holliday SL, Brey RL, Robin BH, Anaya JM, Ahuja S. 2004. Genetic evidence for the common variants/multiple diseases hypothesis. Arthritis Rheum 50:S120.

Humbert P, Dupond JL. 1988. [Multiple autoimmune syndromes]. Ann Med Interne (Paris) 139:159–168.

Jawaheer D, Seldin MF, Amos CI, Chen WV, Shigeta R, Etzel C, Damle A, Xiao X, Chen D, Lum RF, et al. 2003. Screening the genome for rheumatoid arthritis susceptibility genes: a replication study and combined analysis of 512 multicase families. Arthritis Rheum 48:906–916.

Jawaheer D, Seldin MF, Amos CI, Chen WV, Shigeta R, Monteiro J, Kern M, Criswell LA, Albani S, Nelson JL, et al. 2001. A genomewide screen in multiplex rheumatoid arthritis families suggests genetic overlap with other autoimmune diseases. Am J Hum Genet 68:927–936.

Jimenez I, Mora O, Lopez G, Jimenez ME, Zuluga L, Isaza R, Sanchez JL, Uribe CS, Valenzuela CY, Blanco R, Arcos-Burgos M. 1996. Idiopathic epilepsy with generalized tonic clonic seizures in Antioquia Colombia: is the joint Amerindian and Negroid racial admixture the cause of its high prevalence? Biol Res 29:297–304.

Knight JC. 2005. Regulatory polymorphisms underlying complex disease traits. J Mol Med 83:97–109.

Koskenmies S, Widen E, Onkamo P, Sevon P, Julkunen H, Kere J. 2004. Haplotype associations define target regions for susceptibility loci in systemic lupus erythematosus. Eur J Hum Genet 12:489–494.

Kuokkanen S, Gschwend M, Rioux JD, Daly MJ, Terwilliger JD, Tienari PJ, Wikstrom J, Palo J, Stein LD, Hudson TJ, et al. 1997. Genomewide scan of multiple sclerosis in Finnish multiplex families. Am J Hum Genet 61:1379–1387.

Laberge G, Mailloux CM, Gowan K, Holland P, Bennett DC, Fain PR, Spritz RA. 2005. Early disease onset and increased risk of other autoimmune diseases in familial generalized vitiligo. Pigment Cell Res 18:300–305.

Laird NM, Cuenco KT. 2003. Regression methods for assessing familial aggregation of disease. Stat Med 22:1447–1455.

Larsen F, Oturai A, Ryder LP, Madsen HO, Hillert J, Fredrikson S, Sandberg-Wollheim M, Laaksonen M, Harbo HF, Sawcer S, et al. 2000. Linkage analysis of a candidate region in Scandinavian sib pairs with multiple sclerosis reveals linkage to chromosome 17q. Genes Immun 1:456–459.

Lin JP, Cash JM, Doyle SZ, Peden S, Kanik K, Amos CI, Bale SJ, Wilder RL. 1998. Familial clustering of rheumatoid arthritis with other autoimmune diseases. Hum Genet 103:475–482.

Lindqvist AK, Steinsson K, Johanneson B, Kristjansdottir H, Arnasson A, Grondal G, Jonasson I, Magnusson V, Sturfelt G, Truedsson L, et al. 2000. A susceptibility locus for human systemic lupus erythematosus (hSLE1) on chromosome 2q. J Autoimmun 14:169–178.

Ma Y, Ohmen JD, Li Z, Bentley LG, McElree C, Pressman S, Targan SR, Fischel-Ghodsian N, Rotter JI, Yang H. 1999. A genome-wide search identifies potential new susceptibility loci for Crohn's disease. Inflamm Bowel Dis 5:271–278.

Maas K, Chen H, Shyr Y, Olsen NJ, Aune T. 2005. Shared gene expression profiles in individuals with autoimmune disease and unaffected first-degree relatives of individuals with autoimmune disease. Hum Mol Genet 14:1305–1314.

Mellai M, Giordano M, D'Alfonso S, Marchini M, Scorza R, Giovanna Danieli M, Leone M, Ferro I, Liguori M, Trojano M, et al. 2003. Prolactin and prolactin receptor gene polymorphisms in multiple sclerosis and systemic lupus erythematosus. Hum Immunol 64:274–284.

Mori M, Yamada R, Kobayashi K, Kawaida R, Yamamoto K. 2005. Ethnic differences in allele frequency of autoimmune-disease-associated SNPs. J Hum Genet 50:264–266.

Moriuchi J, Ichikawa Y, Takaya M, Shimizu H, Uchiyama M, Sato K, Tsuji K, Arimori S. 1986. Familial Sjogren's syndrome in the Japanese: immunogenetic and serological studies. Clin Exp Rheumatol 4:237–241.

Moroldo MB, Chaudhari M, Shear E, Thompson SD, Glass DN, Giannini EH. 2004. Juvenile rheumatoid arthritis affected sibpairs: extent of clinical phenotype concordance. Arthritis Rheum 50:1928–1934.

Moutsopoulos HM, Webber BL, Vlagopoulos TP, Chused TM, Decker JL. 1979. Differences in the clinical manifestations of sicca syndrome in the presence and absence of rheumatoid arthritis. Am J Med 66:733–736.

Myerscough A, John S, Barrett JH, Ollier WE, Worthington J. 2000. Linkage of rheumatoid arthritis to insulin-dependent diabetes mellitus loci: evidence supporting a hypothesis for the existence of common autoimmune susceptibility loci. Arthritis Rheum 43:2771–2775.

Namjou B, Kelly JA, Kilpatrick J, Kaufman KM, Nath SK, Scofield RH, Harley JB. 2005. Linkage at 5q14.3-15 in multiplex systemic lupus erythematosus pedigrees stratified by autoimmune thyroid disease. Arthritis Rheum 52:3646–3650.

Oertelt S, Kenny TP, Selmi C, Invernizzi P, Podda M, Gershwin ME. 2005. SNP analysis of genes implicated in T cell proliferation in primary biliary cirrhosis. Clin Dev Immunol 12:259–263.

Oturai A, Larsen F, Ryder LP, Madsen HO, Hillert J, Fredrikson S, Sandberg-Wollheim M, Laaksonen M, Koch-Henriksen N, Sawcer S, et al. 1999. Linkage and association analysis of susceptibility regions on chromosomes 5 and 6 in 106 Scandinavian sibling pair families with multiple sclerosis. Ann Neurol 46:612–616.

Parsons JJ. 1968. In: Parson JJ, editor. Antioqueño colonization in Western Colombia. California: Berkeley.

Pearce SH, Merriman TR. 2006. Genetic progress towards the molecular basis of autoimmunity. Trends Mol Med 12:90–98.

Pericak-Vance MA, Rimmler JB, Haines JL, Garcia ME, Oksenberg JR, Barcellos LF, Lincoln R, Hauser SL, Cournu-Rebeix I, Azoulay-Cayla A, et al. 2004. Investigation of seven proposed regions of linkage in multiple sclerosis: an American and French collaborative study. Neurogenetics 5:45–48.

Prahalad S, Shear ES, Thompson SD, Giannini EH, Glass DN. 2002. Increased prevalence of familial autoimmunity in simplex and multiplex families with juvenile rheumatoid arthritis. Arthritis Rheum 46:1851–1856.

Price P, Witt C, Allcock R, Sayer D, Garlepp M, Kok CC, French M, Mallal S, Christiansen F. 1999. The genetic basis for the association of the 8.1 ancestral haplotype (A1, B8, DR3) with multiple immunopathological diseases. Immunol Rev 167:257–274.

Priori R, Medda E, Conti F, Cassara EA, Danieli MG, Gerli R, Giacomelli R, Franceschini F, Manfredi A, Pietrogrande M, et al. 2003. Familial autoimmunity as a risk factor for systemic lupus erythematosus and vice versa: a case-control study. Lupus 12:735–740.

Reveille JD, Wilson RW, Provost TT, Bias WB, Arnett FC. 1984. Primary Sjogren's syndrome and other autoimmune diseases in families. Prevalence and immunogenetic studies in six kindreds. Ann Intern Med 101:748–756.

Sale MM, FitzGerald LM, Charlesworth JC, Bowden DW, Rich SS. 2002. Evidence for a novel type 1 diabetes susceptibility locus on chromosome 8. Diabetes 51(Suppl 3):S316–S319.

Schmidt MB. 1926. Eine biglandulare Erkrankung (Nebennieren und Schilddrüse) bei Morbus Addisonii. Verh Dtsch Ges Pathol 21:212–221.

Serrano NC, Millan P, Paez MC. 2006. Non-HLA associations with autoimmune diseases. Autoimmun Rev 5:209–214.

Serrano NC, Paez C, Correa PA, Anaya JM. 2004. Endothelial nitric oxide synthase gene polymorphism is associated with systemic lupus erythematosus. J Rheumatol 31:2163–2168.

Silman AJ, MacGregor AJ, Thomson W, Holligan S, Carthy D, Farhan A, Ollier WE. 1993. Twin concordance rates for rheumatoid arthritis: results from a nationwide study. Br J Rheumatol 32:903–907.

Sloka S. 2002. Observations on recent studies showing increased co-occurrence of autoimmune diseases. J Autoimmun 18:251–257.

Steinberg AD, Talal N. 1971. The coexistence of Sjogren's syndrome and systemic lupus erythematosus. Ann Intern Med 74:55–61.

Tait KF, Marshall T, Berman J, Carr-Smith J, Rowe B, Todd JA, Bain SC, Barnett AH, Gough SC. 2004. Clustering of autoimmune disease in parents of siblings from the type 1 diabetes Warren repository. Diabet Med 21:358–362.

Tomer Y, Ban Y, Concepcion E, Barbesino G, Villanueva R, Greenberg DA, Davies TF. 2003. Common and unique susceptibility loci in Graves and Hashimoto diseases: results of whole-genome screening in a data set of 102 multiplex families. Am J Hum Genet 73:736–747.

Tsao BP, Cantor RM, Grossman JM, Kim SK, Strong N, Lau CS, Chen CJ, Shen N, Ginzler EM, Goldstein R, et al. 2002. Linkage and interaction of loci on 1q23 and 16q12 may contribute to susceptibility to systemic lupus erythematosus. Arthritis Rheum 46:2928–2936.

Vaidya B, Imrie H, Perros P, Young ET, Kelly WF, Carr D, Large DM, Toft AD, Kendall-Taylor P, Pearce SH. 2000. Evidence for a new Graves disease susceptibility locus at chromosome 18q21. Am J Hum Genet 66:1710–1714.

Vyse TJ, Todd JA. 1996. Genetic analysis of autoimmune disease. Cell 85:311–318.

Wandstrat A, Wakeland E. 2001. The genetics of complex autoimmune diseases: non-MHC susceptibility genes. Nat Immunol 2:802–809.

Winchester R. 1992. Systemic lupus erythematosus. New York: Churchill Living Stone.

Yamada R, Ymamoto K. 2005. Recent findings on genes associated with inflammatory disease. Mutat Res 573:136–151.

